# Simultaneous Determination of 15 Mycotoxins in Aquaculture Feed by Liquid Chromatography–Tandem Mass Spectrometry

**DOI:** 10.3390/toxins14050316

**Published:** 2022-04-28

**Authors:** Beatriz Albero, María Luisa Fernández-Cruz, Rosa Ana Pérez

**Affiliations:** Department of Environment and Agronomy, National Institute for Agricultural and Food Research and Technology (INIA), Spanish National Research Council (CSIC), 28040 Madrid, Spain; albero@inia.csic.es

**Keywords:** fish feed, multiresidue, enniatins, beauvericin, fumonisin, analysis

## Abstract

The use of plant-based fish feed may increase the risk of contamination by mycotoxins. The multiresidue analysis of mycotoxins in fish feed presents many difficulties due to the complexity of the matrix, the different characteristics of the compounds, and their presence in highly different concentration levels. The aim of this study was to develop a selective, sensitive, and efficient analytical method for the simultaneous determination of 15 mycotoxins (regulated and emerging mycotoxins) in aquaculture feed by LC-MS/MS. Sample extraction was performed with ultrasonic assistance, and different cleanup strategies were evaluated. The optimized method was composed by ultrasound-assisted extraction (two cycles, 55 °C, 20 min), followed by cleanup using a Captiva EMR Lipid cartridge. Then, nine commercial samples of aquaculture fish feed were analyzed. Eight of the 15 target mycotoxins were detected in the samples. Results showed that two enniatins (EENB and ENNB1), beauvericin, and fumonisin B2 were detected in all samples. These results show the multi-mycotoxin contamination of fish feed, highlighting the need to improve current knowledge on the occurrence and toxicity of mycotoxins in fish feed, mainly the emerging ones.

## 1. Introduction

The world population is growing at a rate where, by 2050, over 9.7 billion people will have to be fed, and a significant increase in food production (1.8% every year) will be necessary [1]. Therefore, food supply will present a growing challenge in the next three decades. Fish consumption has been increasing worldwide; consequently, some concerns have begun to emerge, primarily regarding the quality of fish available on the market. Global fish production is estimated to have reached about 179 million tons in 2018, and aquaculture accounted for 46% of the total production and 52% of fish for human consumption [2]. A growing share of fishmeal and fish oil, 25–35%, is produced from the byproducts of fish processing.

The limited availability of protein sources from marine origin has compelled the development of fish feeds with high contents of plant-based ingredients, such as plant meals and vegetable oils, and a low inclusion level of marine ingredients [3]. Plant-based feeds can introduce contaminants, which could be relevant in aquaculture, not previously associated with fish farming when traditional feed ingredients were of marine origin. Thus, concerns about potential risks to human and animal welfare have led to increased interest in the evaluation of contaminants in aquaculture feed formulations. Mycotoxins are included as potential contaminants in plant-based fish feed as natural contaminants in cereals and oilseeds. Mycotoxins are a broad group of toxic secondary metabolites of fungi. While only a small number of plant pathogenic fungal species are known to produce mycotoxins, most spoilage fungi secrete a range of toxic metabolites. The toxinogenic fungal genera most commonly isolated from food and feed products are *Aspergillus, Fusarium*, and *Penicillium* [4]; they can be categorized into two groups: field fungi (e.g., *Fusarium* spp.) that access the crop during its growth and storage fungi (e.g., *Aspergillus* spp., *Penicillium* spp.) that mostly contaminate the crop post harvest [5]. Gonçalves et al. [6] concluded that, due to the use of increasing levels of plant meals in fish feeds, as well as the proliferation of mycotoxins due to climate change, more studies on the impact of mycotoxins and metabolites on farmed species together with a risk assessment for mycotoxin-contaminated fish feed are necessary.

Most of the earlier studies evaluating mycotoxins in fish feeds mainly focused on aflatoxin (AF) occurrence, and other mycotoxins have only recently been analyzed [6]. Thus, AFs, trichothecenes, fumonisins of the B series, ochratoxins, and zearalenone (ZEN) have been considered as the mycotoxins that pose the greatest potential risk to human and animal health due to their prevalence in feeds and their effects on livestock health [4]. Additionally, there is a new group of emerging mycotoxins, such as beauvericin (BEA) or enniatins (ENNs), produced by *Fusarium* spp., for which information on their occurrence is scarce, especially in aquaculture feeds [6].

Multi-toxin contamination of fish feed might result from the contamination of plant-based ingredients, because fungal genera produce more than one mycotoxin simultaneously (e.g., *Fusarium* spp.) or the feed can be contaminated by different fungi. Currently, European legislation only considers mycotoxin mono-exposure data [7,8] and multi-mycotoxin exposure is not envisaged, although it is stated that adverse effects on animal health and performance can be additive and/or synergistic [4]. The determination of the contamination of fish feed by mycotoxins is essential to improve the current state of knowledge about the quality and toxicity of products intended for livestock feed.

Due to a growing interest in the determination of mycotoxins in food and their effects, some multiresidue methods including a large number of mycotoxins have been developed focusing on raw cereals, whereas data on the performance characteristics in other matrices such as fish feed are scarce. Thus, Tebele et al. [9] developed a method for the validation and quantification of 22 mycotoxins in household staple cereals (maize, sorghum, and wheat) by LC-MS/MS, in which the extraction was carried out with ACN/water/acetic acid (79:20:1 *v*/*v*/*v*) for 90 min at 180 rpm using a mechanical shaker. Varga et al. [10] developed a multitarget UHPLC-MS/MS method for the determination of 191 fungal metabolites in almonds, hazelnuts, peanuts, and pistachios, and apparent recoveries between 80% and 120% were obtained for about half of the analytes, although recoveries were lower than 38% for some analytes. Kim et al. [11] developed an analytical method for the determination of emerging mycotoxins in cereal and cereal-based products by UHPLC-MS/MS. Rausch et al. [12] developed a method for the determination of 38 native and modified mycotoxins in cereals. Considering that fish feed is a very complex sample with a high fat content, multiresidue analysis of mycotoxins in fish feed requires the development of specific analytical methodology.

Although information regarding the occurrence and effects of mycotoxins in fish feed and fish is still scarce, in the last 5 years, some reviews focusing on these issues were published [5,6,13]; however, emerging mycotoxins were not included in these reviews. As result of the scarce information about the emerging mycotoxins, such as BEA and ENNs, concern about the risk of exposure to these toxins has been addressed by the European Commission, requesting the European Food Safety Authority for a scientific opinion on their risk to human and animal health [3].

The multiresidue analysis of mycotoxins in fish feed presents many difficulties due to the complexity of the matrix, the different characteristics of the compounds, and their presence in very different concentration levels. Many of the methods reported for the detection of mycotoxins are focused on cereals and in regulated mycotoxins, with only a few methods combining mycotoxins from different families to assess the co-occurrence of mycotoxins in other matrices [4]. Scarpino et al. [14] developed and compared two multiresidue methods for the determination of emerging and biologically modified (i.e., 15-acetyldeoxynivalenol, 15-a-DON) mycotoxins in maize and wheat. They pointed out that there is an urgent need to develop accurate, precise, and sensitive multiresidue analytical methods for the analysis of regulated and emerging mycotoxins in food and feed matrices in order to acquire data on their co-occurrence. Juan et al. [15] developed a QuEChERS method to analyze the presence of 22 mycotoxins (including emerging mycotoxins) in 122 Tunisian marketed feed samples, most commonly finding the co-occurrence of five different mycotoxins (26%), with up to eight mycotoxins found in 5% of samples.

Liquid chromatography–tandem mass spectrometry (LC-MS/MS) has become the analytical technique most used for the determination of mycotoxins, although enzyme immunosorbent analysis has also been applied [4]. In order to minimize the matrix effects that have a detrimental effect on LC-MS/MS, intensive cleanup strategies have been adopted such as sequential solid-phase extraction (SPE), QuEChERS-like methods, or the dilute-and-shoot method. The latter offers the opportunity to reduce or even avoid sample cleanup, but this could undermine the quantification of mycotoxins [4,14].

Accordingly, the main objective of this study was to develop a selective, sensitive, and efficient analytical method for the simultaneous determination of 15 mycotoxins (regulated and emerging mycotoxins) in aquaculture feed by LC-MS/MS. This paper includes the most important mycotoxins found in fish feed and its raw materials: aflatoxins (AFB1, AFB2), fumonisins (FB1, FB2), ochratoxin A (OTA), nivalenol (NIV), deoxynivalenol (DON), 3-acetyldeoxynivalenol (3-a-DON), 15-a-DON, ZEN, and emerging *Fusarium* mycotoxins (BEA, ENNA, ENNA1, ENNAB, and ENNAB1). The validated method was used to monitor these contaminants in aquaculture feed for trout. Thus, this study reports data on potential multi-mycotoxin contamination, as well as the levels of emerging mycotoxins for trout feeding. As indicated above, only a few studies have reported the analysis of a wide set of mycotoxins in fish feed including emerging mycotoxins.

## 2. Results and Discussion

### 2.1. HPLC-MS/MS Determination

The chromatographic response of individual mycotoxins was very different. Thus, the relative proportion of these in the working mixture solution was established considering their individual chromatographic response.

**Figure 1:**
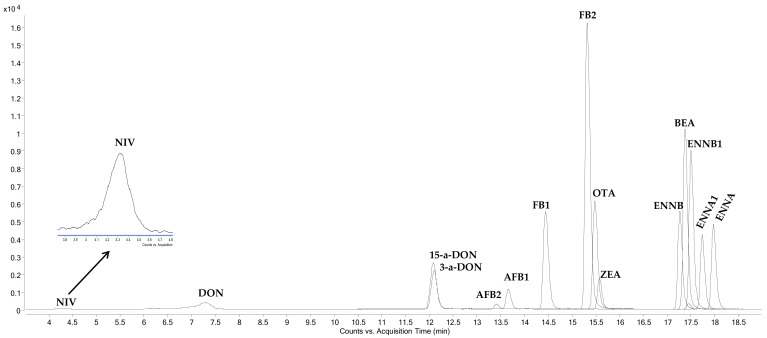
MRM chromatogram of a blank fish feed extract spiked at a concentration range of 2 to 300 ng/mL.

Therefore, the six mycotoxins with the lowest concentration in the working mixture were AFB2, AFB1, BEA, ENNB, ENNA, and ENNA1, followed by ENNB1 and OTA (10× higher), 15-a-DON and 3-a-DON (25× higher), FB2, FB1, and DON (100× higher), and ZEN and NIV (150× higher). Figure 1 shows the chromatogram obtained for a blank feed sample spiked with the mixture of mycotoxins in the relative proportion selected. Note the low response of NIV despite being one of the compounds that had the highest concentration in the mixture. Fish feed extracts were obtained in acetonitrile/H_2_O/acetic acid (79:20:1, *v*/*v*/*v*), evaporated to dryness, and reconstituted in the initial conditions of the mobile phase (H_2_O/methanol 80:20, *v*/*v* containing 3 mM ammonium formate and 0.1% (*v*/*v*) formic acid), as described by Dagnac et al. [16] for mycotoxins in maize. However, in these conditions, feed fish extracts showed turbidity that did not disappear after filtration through 0.22 μm or after increasing the proportion of organic solvent in the extract. For this reason, the final extract was not evaporated to change the solvent to methanol as applied by other authors [10,14]. The proportions of 79% and 50% of organic solvent in the final extract were compared, and a high increase in the chromatographic response was observed with 50% acetonitrile. Thus, this was the solvent mixture selected for the chromatographic analysis.

### 2.2. Optimization of the Extraction Procedure

The mycotoxins that were chosen for evaluation in fish feed were both the regulated mycotoxins commonly found in cereals and the main emerging mycotoxins. The selective extraction of analytes from fish feed is a very complicated task, and a cleanup step is necessary to minimize the co-elution of matrix components in the ionization process in the mass spectrometer.

In a first approach, a method based on ultrasound-assisted matrix solid-phase dispersion (UA-MSPD) was assayed. This technique has been successfully applied in our laboratory for the multiresidue determination of emerging contaminants in vegetables and cereals [17,18]. In the selection of the extraction solvent, it was taken into account that polar toxins such as FBs require aqueous solutions, while organic solvents are necessary for the extraction of hydrophobic toxins such as aflatoxins. Although methanol was initially considered as the extraction solvent to have a final extract similar to the chromatographic mobile phase, acetonitrile was selected because it was reported that it improved extraction efficiency and that the acidification of the extraction solvent improves the recovery of acidic mycotoxins such as fumonisins or OTA [16]. The use of the mixture acetonitrile/H_2_O/acetic acid (79:20:1 *v*/*v*/*v*) was reported as adequate for the extraction of mycotoxins in cereal and related foodstuffs [19] and in animal feed [4]. Therefore, it was selected as extraction solution for the subsequent UA-MSPD procedure. In a glass mortar, 2 g of ground sample was mixed with quartz sand (2 g) and C18 (1 g) using a glass pestle. The mixture was then transferred to a glass column before adding 7 mL of the extraction solution. The columns were placed in an ultrasonic water bath at room temperature for a 15 min sonication cycle. Then, the extracts were collected under vacuum, and the procedure was repeated with another 6 mL of the extraction solvent. The combined extracts were concentrated to 0.5 mL and diluted with H_2_O/methanol (80:20, *v*/*v*) to 2 mL. Very dirty extracts were obtained, necessitating a cleanup step. Two different purification strategies were assayed: dispersive solid-phase extraction (dSPE) with PSA or liquid–liquid extraction (LLE) with hexane. Hexane was employed to eliminate fat particles in the determination of mycotoxins in cereals and cereal-based food [20], as well as the emerging *Fusarium* mycotoxins in feed and fish from aquaculture [21]. The use of hexane in the cleanup of extracts was an interesting alternative because it is less expensive than PSA sorbent. Thus, on the one hand, different amounts of PSA (50, 80, or 100 mg) were assayed for the dSPE cleanup of 1 mL extract. Clear extracts were only obtained with 100 mg of PSA. Nevertheless, the recoveries obtained for many of the target mycotoxins were very low (Table 1). On the other hand, the purification by LLE of 1 mL of extract with the same amount of hexane was assayed. Thus, the mixture was shaken, and, after centrifugation, the hexane phase was discarded. The extracts obtained were clear and could be chromatographically evaluated, but the recoveries of the mycotoxins were still very low, except for NIV, DON, 15-a-DON, AFB2, and AFB1 (see Table 1).

The effect on the recoveries of a defatting step with hexane before the UA-MSPD, which would simplify the extraction method, was evaluated. Defatting was carried out adding 3 mL of hexane to the column and sonicating 5 min. Hexane was removed by vacuum before adding 4 mL of the extraction solution to perform the ultrasound-assisted extraction of the samples as described above. The final extracts were concentrated to dryness, diluted with H_2_O/methanol (80:20, *v*/*v*), and filtered before the chromatographic analysis. This change yielded an increase in the recoveries of all mycotoxins with values from 39% to 111%, except for ENNA and BEA with recoveries of 25% and 10.5%, respectively (see Table 1). Defatting of extracts with hexane could give losses in the range of 31–62% for BEA and ENNAs [22]. Although ZEN is another rather lipophilic compound and its recovery could be affected by hexane cleanup [23], Boevre et al. [20] reported that only a loss <2.5% of ZEN was observed when the extraction was carried out in combination with hexane. Then again, fish feed has a very high fat content, and the volume of hexane needed in the defatting step is proportionally higher than for cereal samples.

In order to improve the recoveries of the target mycotoxins in fish feed, UA-MSPD was replaced by ultrasound-assisted extraction (UAE), carrying out the extraction in absence of sorbents, followed by the cleanup of the extracts employing a Captiva EMR Lipid cartridge. The preliminary assays showed good recoveries of all the compounds, except for FB1 and FB2. The use of the Captiva EMR Lipid cartridge has been previously reported as a successful tool for the analysis of pesticides in fatty vegetable matrices [24] and PAHs in smoked food of animal origin [25]. For mycotoxin analysis, the Captiva EMR Lipid cartridge has been reported for the determination of mycotoxins in cheese [26], infant formula [27], and human plasma [28]. Nakhjavan et al. [29] employed this lipid removal cartridge instead of dSPE, after QuEChERS extraction, for the determination of mycotoxins in feed products, although emerging mycotoxins were not included. Therefore, to the best of our knowledge, this is the first time that lipid removal cartridges have been used for the multiresidue analysis of emerging mycotoxins in fish feed.

Taking into account the promising preliminary results with the Captiva EMR Lipid cartridge, the extraction method was optimized, validated, and applied to the multiresidue analysis of mycotoxins in fish feed.

**Figure 2:**
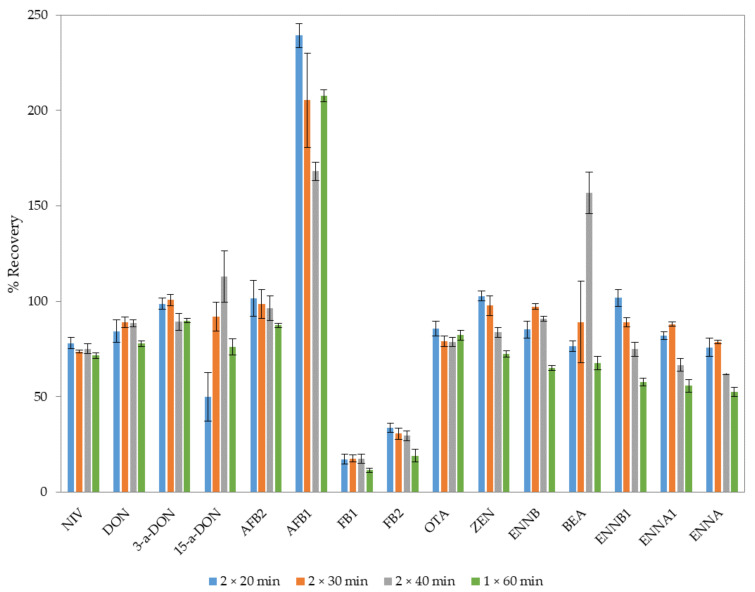
Effect of the sonication cycles on the extraction of mycotoxins from fish feed spiked at the high concentration level (*n* = 3).

In order to enhance the extraction efficiency, particularly of the four compounds with low recoveries, the effect of some parameters was evaluated, such as temperature and extraction time, which are the main parameters that usually affect the efficiency of the UAE step. Thus, two sonication cycles from 20 to 40 min and a single 60 min sonication cycle with acetonitrile/H_2_O/acetic acid (79:20:1 *v*/*v*/*v*) were assayed and compared. Results are summarized in Figure 2.

In general, similar recoveries were obtained after two 20 and 30 min sonication cycles, except for 15-a-DON, and these were similar or lower for longer sonication cycles. In addition, UAE with two 30 min sonication cycles provided higher extraction yields than performing one sonication cycle (60 min). The recovery for AFB1 was very high in all the assays. Stroka et al. [30] investigated various extraction solvents for the analysis of AFB1 in different food and feed matrices, and they found that the use of aqueous acetonitrile could absorb significant amounts of water from the extraction solution, resulting in recoveries that were too high.

**Figure 3:**
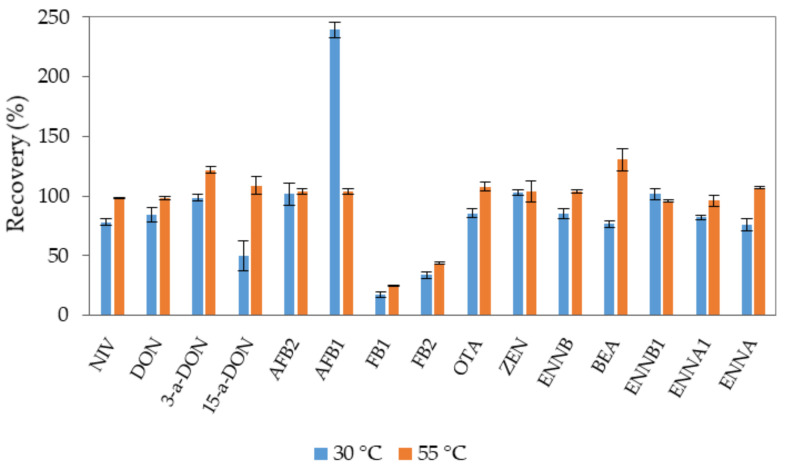
Effect of temperature when 2 × 20 min UAE cycles were carried out in the extraction of mycotoxins, when fish feed was spiked at the high level (*n* = 3).

According with the results of the extraction time, two extractions of 30 min could be adequate. Nevertheless, in order to minimize the processing time, the effect of temperature (30 °C vs. 55 °C) after two UAE cycles of 20 min was evaluated. At the higher temperature, quantitative recoveries of 15-a-DON were obtained, and the recovery of AFB1 at 55 °C was 104% instead of 239% (see Figure 3). Therefore, 20 min at 55 °C was selected for the UAE step.

### 2.3. Method Validation

The mass spectrometric response of target analytes may be affected by the coelution of matrix components; therefore, the matrix effect was evaluated preparing a set of six standard solutions in the range from 0.5 to 10 ng/mL (for the compounds at the lowest concentration in the mixture) in acetonitrile/formic acid 0.1% (1:1, *v*/*v*). Another set of calibration standards were prepared by spiking blank fish feed extracts in the same concentration range. The slopes obtained, for both sets of calibration standards, by plotting concentration against peak area following linear regression analysis, were compared. The percentage matrix effects were determined by the following equation: % matrix effect = ((matrix slope − solvent slope)/solvent slope) × 100 [31]. The response of FB2, OTA, and ZEN was not affected by the presence of matrix components, whereas, for most of the other mycotoxins, a matrix-induced signal suppression (−20% to −52%) was observed. To avoid matrix effects and achieve adequate quantitation of mycotoxins in fish feed, the standard addition method was used.

The optimized extraction method (see Section 4) was validated in terms of linearity accuracy, precision, and limits of detection and quantitation. The linearity of the method was evaluated injecting six spiked fish feed blank extracts in the range from 0.05 to 10 ng/mL, which was equivalent to 0.7 to 140 μg/kg (for the compounds at the lowest concentration in the mixture). Moreover, a good linearity was obtained, with correlation coefficients ≥0.996 for all the studied compounds.

The precision of the method was evaluated in terms of repeatability (intra-day precision) and reproducibility (inter-day precision). Repeatability was evaluated by analyzing seven replicates of a blank extract spiked at the lower level (from 1 to 150 μg/kg) within a given day, and the precision was <6%, expressed as RSD. Reproducibility was evaluated by determining, on three different days, three replicates at the highest and lowest spiking levels, and RSDs < 18% for all the compounds were obtained.

The accuracy of the method was evaluated performing the recovery of the target analytes from fish feed spiked at three different levels. Recovery results, obtained using matrix-matched standards, are shown in Table 2. In general, good recoveries were obtained for the target mycotoxins, except for FB1 and FB2. The method developed by Varga et al. [10] for the determination of a broad number of mycotoxins in different types of nuts reported recoveries between 80% and 120% for about half of the analyte–matrix combinations, but compounds such as NIV, DON, FB1, and FB2 showed mean recoveries of 19%, 33%, 38%, and 23%, respectively. Mwihia et al. [32] reported recoveries of 52% and 57% for FB1 and FB2 respectively in fish feed from Kenia. The spiking level employed (200 μg/L of each mycotoxin) was much higher than that assayed in the present study (from 7 to 71 μg/L). Recently, Konak et al. [33] developed a method for the simultaneous analysis of antibiotics and mycotoxins in feed, which included FB1 and FB2, and their results showed that the extraction procedure was not suitable for these mycotoxins. The analysis of mycotoxins in fish feed reported by Tolosa et al. [17] only determined five emerging mycotoxins with recoveries from 88% to 104%. In their method, 5 g of feed was extracted with 50 mL of acetonitrile by Ultra-Turrax homogenization followed by centrifugation and cleanup of the supernatant using C18 cartridges. Taking into account the reported works, the developed method allowed the analysis of a broader number of mycotoxins with similar recoveries to those reported by other authors.

The limits of detection (LODs) and limits of quantification (LOQs) of the developed method were calculated by analyzing seven replicates of fish feed extracts spiked at levels from 0.7 to 105 μg/kg, considering the proportion of mycotoxin in the mixture. The LOD and LOQ for NIV had to be determined by employing a blank feed extract spiked at a higher level. The LOD and LOQ values were obtained following the t99sLLMV approach developed by EPA [34], and the results are summarized in Table 2. The LOQ values ranged from 0.16 to 59 μg/kg (except for NIV that was 180 μg/kg). The LOQs achieved in this study were much lower, except for NIV, than those achieved by Mwihia et al. [32] for the determination of 40 mycotoxins in fish feeds in Kenya. The LOQs for emerging mycotoxins, 0.16–0.7 μg/kg, were in the same range as those reported by Tolosa et al. [21] in fish feed, but lower than those achieved in cereals and derivatives [11].

According with these results, the present study provides a multiresidue method suitable for the simultaneous detection and quantification of 15 mycotoxins in fish feed with good LOQs, taking into account the limits reported for other food commodities (see Table 3).

### 2.4. Real Samples

Once the analytical method was optimized and validated, it was applied for the analysis of nine fish feed samples. Table 4 summarizes the levels of mycotoxins detected in the samples analyzed. Results showed that eight of the 16 target mycotoxins were detected in at least two of the samples analyzed. Three of the emerging mycotoxins, ENNB, ENNB1, and BEA were detected in all samples at quantifiable levels, whereas FB2 was detected in all samples, but only one was above the LOQ. Although the levels of enniatins found in fish feed were low, this issue should be taken into account. A recent study to determine the toxicity of these mycotoxins in the fish cell line RTGill-W1 showed that ENNA, ENNA1, ENNB1, BEA, and ZEA were highly toxic [36]. The presence of enniatins and BEA in feed fish from Valencian hatcheries was evaluated, and all the samples analyzed were contaminated with enniatins, while 95% were contaminated with BEA [21]. The levels found were in the range of 0.1 to 10 μg/kg. These results are in accordance with those found in the present study, although BEA was found at levels up to 30 μg/kg in a sample in which eight of the studied mycotoxins coexisted, at levels above the LOQ. In another study, the levels of emerging mycotoxins in feeds formulated with high levels of plant-derived ingredients were evaluated [3]. Results obtained showed that these mycotoxins were detected in all the feeds. They found the highest values for BEA (80.4 μg/kg), followed by ENNB (8.0–32.8 μg/kg), ENNB1 (2.4–10.9 μg/kg), and ENNA1 (up to 3.8 μg/kg), while the level of ENNA was below the LOQ (<1 μg/kg) in all cases.

In a review on mycotoxins in aquaculture, the authors distinguished between the mycotoxins analyzed before and after 2012 [6]. Before 2012, AFs (mostly AFB1) and in, some cases, ZEN and OTA were the main target mycotoxins, according to data reported on terrestrial livestock feed samples. After 2012, other mycotoxins started to be reported, probably as a reflection of an increasing awareness of mycotoxins in aquaculture and as a result of the development of new analytical methods to determine mycotoxins. In our samples, AF contamination was not detected. The presence of AFs in fish feed could be indicative of inadequate storage conditions of raw materials or feeds [6].

Results reported by Fegan and Spring [37], regarding the presence of AF, T-2, ZEN, and OTA in nine fish feed samples collected in Thailand, showed predominantly ZEN contamination in all samples, at levels ranging from 36.2 to 118.5 μg/kg, in addition to OTA contamination (2.3 to 7.7 μg/kg). The presence of ZEN in trout feed collected from three farms was detected at levels of 10 and 82 μg/kg [38]. In Atlantic salmon (*Salmo salar*) feeds, the most representative mycotoxins found of the 18 evaluated were FBs (112–148 μg/kg) and DON (19–23 μg/kg) [39]. In our study, ZEN was detected in only two of the nine samples evaluated, being quantified only in one sample at a level of 121 μg/kg; DON was not detected in any samples, while FB1 and FB2 were detected in 56% and 100% of the samples, respectively.

## 3. Conclusions

A method, based on UAE and SPE cleanup using the Captiva EMR Lipid cartridge, was successfully developed for the analysis of 15 mycotoxins, belonging to different classes, in feed fish. The method showed satisfactory recovery values, except for FBs, with LODs between 0.05 and 54 μg/kg. After method validation, the procedure was applied to analyze nine rainbow trout feed samples, and eight of the target mycotoxins were detected in the samples. ENNB, ENNB1, BEA, and FB2 were detected in all samples. These results show the multi-mycotoxin contamination of fish feed, highlighting the need to improve current knowledge on the occurrence and toxicity of mycotoxins in fish feed, mainly the emerging ones. Further studies to improve knowledge on the bioaccumulation profile and toxicological effects of mycotoxins in fish resulting from feed contamination are required.

## 4. Materials and Methods

### 4.1. Reagents and Materials

All of the mycotoxin standards were purchased from Sigma Aldrich (Steinheim, Germany). Individual stock solutions were prepared at 1–10 mg/mL in dimethyl sulfoxide (DMSO) except for DON, 3-a-DON, 15-a-DON, and BEA that were prepared in methanol. Working standard solutions containing all analytes were prepared by an appropriate dilution of the stock standard solutions with methanol and stored in amber vials at −20 °C. HPLC-grade acetonitrile and methanol were purchased from Sigma Aldrich (Steinheim, Germany), while hexane, GC residue analysis grade, was obtained from Scharlab (Barcelona, Spain). Ammonium formate, 99% purity, was purchased from Sigma Aldrich (Steinheim, Germany). Formic acid and acetic acid were acquired from Honeywell Fluka (Seelze, Germany). Bulk Extrabond^®^ C18 and PSA were obtained from Scharlab (Barcelona, Spain). Captiva EMR Lipid (3 mL) cartridges were obtained from Agilent Technologies (Santa Clara, CA, USA). Ultrahigh-purity water was obtained from a MilliQ water purification system (Millipore, Spain).

### 4.2. Samples

Nine rainbow trout feed samples from two different commercial brands were provided by Escuela Técnica Superior Ingenieros de Montes, Universidad Politécnica, Madrid, Spain. Feed pellets had sizes between 0.1 and 1.9 mm. The feed sample employed in the optimization of the analytical method included, as nutrient content, crude protein (44%), crude fats (23%), crude fiber (2.3%), ashes (8.4%), and total phosphorus (1.19%). This fish feed was composed of fish meal and fish oil, together with wheat flour, poultry blood meal, field peas, rapeseed meal, and sunflower cake, among other ingredients.

### 4.3. Sample Preparation

Ground fish feed (1 g) was placed in a 20 mL glass column with a cellulose frit at the bottom and closed with a stopcock. UAE was carried out with 4 mL of acetonitrile/H_2_O/acetic acid (79:20:1, *v*/*v*/*v*) in an ultrasonic water bath (360 W, 50–60 Hz) at 55 °C for 20 min. The extracts were collected in graduated glass tubes by means of a vacuum manifold. An additional sonication cycle was carried out with 4 mL of the extraction solution. The volume was brought to 7 mL. A 2 mL aliquot was cleaned up by employing the Captiva EMR Lipid cartridge. The purified extract was diluted (1:1) with acetonitrile/H_2_O (20:80) and filtered through a 0.2 μm nylon filter before chromatographic analysis.

### 4.4. LC-MS/MS Analysis

Analyses were performed on an Agilent 1200 LC system (Waldbronn, Germany). A Kinetex XB-C18 (100 mm × 3 mm i.d., 2.6 μm particle size) analytical column with a C18 security guard cartridge from Phenomenex (Torrance, CA, USA) was employed for the chromatographic separation of the analytes. Chromatographic separation was performed at a flow rate of 0.2 mL/min with the column at room temperature, following the method published by Dagnac et al. [16] with slight modifications. The mobile phase was a time-programmed gradient using H_2_O (eluent A) and methanol (eluent B), both containing 3 mM ammonium formate and 0.1% (*v*/*v*) formic acid. Gradient elution was started isocratically with 80% A for 1 min. Then, B was linearly increased to 100% within 9 min and kept constant for 4 min. Finally, B was decreased to 20% in 10 min and equilibrated for 2 min. A post-run time of 4 min was allowed before the next injection.

Mass spectrometry was performed with an Agilent 6420 triple-quadrupole mass spectrometer (Waldbronn, Germany) equipped with an electrospray ionization interface, operating in positive and negative ion mode. The following mass spectrometer parameters were set: drying gas temperature of 300 °C, drying gas flow rate of 9 L/min, nebulizer gas pressure of 35 psi, and capillary voltage of 3500 V.

For both identification and quantification of the analytes, one precursor ion and two product ions for each target compound were selected to work in multiple reaction monitoring (MRM) mode. The precursor and product ions with their optimal collision energies and fragmentor voltages, are summarized in Table 5. Analytes were confirmed by their retention time and the identification of quantifier and qualifier transitions. Retention times had to be within ±0.2 min of the expected time, and qualifier-to-quantifier ratios had to be within a 20% range for positive confirmation.

## Figures and Tables

**Table 1:** Comparison of the recoveries of mycotoxins by UA-MSPD and different cleanup strategies. Each assay was performed with three replicates.

Compounds	After Extraction				Before Extraction	
	dSPE-PSA		LLE-Hexane		Hexane Defatting	
	Mean	SD	Mean	SD	Mean	SD
NIV	97.6	7.0	82.8	8.3	80.2	3.3
DON	104.0	4.9	85.8	7.1	90.2	2.6
3-a-DON	97.5	2.4	85.7	2.9	110.9	14.0
15-a-DON	48.1	3.1	43.3	2.1	89.2	1.9
AFB2	75.9	5.3	74.2	2.5	97.4	5.5
AFB1	72.7	4.1	63.2	0.9	96.4	2.6
FB1	14.9	5.4	12.8	0.9	44.7	2.6
FB2	42.0	3.2	4.3	1.1	43.7	3.5
OTA	12.9	0.5	14.4	2.0	60.4	4.2
ZEN	1.2	0.3	6.0	0.8	42.5	0.3
ENNB	0.8	0.3			73.6	10.5
BEA	1.2	1.1			2.3	0.1
ENNB1	0.8	0.3	2.3	1.7	39.3	2.4
ENNA1	2.0	0.9	1.2	0.3	33.7	3.3
ENNA	1.6	0.6	14.6	0.3	24.7	2.7

**Table 2:** Recoveries (*n* = 3), linearity range, quantification limits (LOQs), and detection limits (LODs) of mycotoxins in fish feed.

	Linearity Range μg/kg	Spiking Level μg/kg	Recovery (%)		Spiking Level μg/kg	Recovery (%)		Spiking Level μg/kg	Recovery (%)		LOD	LOQ
			Mean	SD		Mean	SD		Mean	SD	μg/kg	μg/kg
NIV	200–21,000	150			750	76	9	1500	88	1	54	180
DON	70–1400	100	104	3	500	91	4	1000	102	2	11	32
3-a-DON	18–3500	25	127	8	125	91	6	250	122	5	3	9
15-a-DON	18–3500	25	90	7	125	91	7	250	109	8	3	9
AFB2	0.7–140	1	113	8	5	87	6	10	94	3	0.2	0.6
AFB1	0.7–140	1	102	10	5	92	6	10	95	4	0.1	0.4
FB1	70–14,000	100	10	2	500	22.	7	1000	25	1	6	18
FB2	70–14,000	100	21	6	500	35	7	1000	44	1	9	27
OTA	7–1400	10	100	16	50	79	6	100	108	3	2	6
ZEN	105–21,000	150	119	18	750	51	5	1500	104	9	20	59
ENNB	0.7–140	1	104	5	5	75	6	10	104	2	0.08	0.25
BEA	0.7–140	1	118	9	5	96	6	10	130	9	0.05	0.16
ENNB1	7–1400	10	64	6	50	70	7	100	96	1	0.1	0.3
ENNA1	0.7–140	1	66	5	5	72	9	10	96	5	0.2	0.5
ENNA	0.7–140	1	58	6	5	66	9	10	107	1	0.2	0.7

**Table 3:** Summary of LOQ values (μg/kg) reported for the target mycotoxins in feed samples.

Mycotoxin	Matrix						
	Cereals ^a^	Maize Silage ^b^	Animal Feed ^c^	Cereal and Derivatives ^d^	Fish Feed ^e^	Fish Feed ^f^	Fish Feed ^g^
NIV			100		134		180
DON	60–214	57	100		135		32
3-a-DON	0.15–0.4	2.6 ^h^	50		120		9
15-a-DON	59.6–110.8	2.6 ^h^			409		9
AFB1	0.04–12.3	0.08–0.5	1		49		1.2
AFB2	0.04–9.3		1		60		0.4
FB1	0.03–22.8	2.9	50		209		18
FB2	1.2–20.8	6.4	50		230		27
OTA	0.3–1.9	0.48	5		91		6
ZEN	0.05–11.4	5.6	10		127		59
ENNB		0.08		0.2	129	0.1	0.25
BEA				6.1	53	0.1	0.16
ENNB1		0.08		7.2	43	0.1	0.3
ENNA1				4.1	45	0.25	0.5
ENNA					1.3	87	0.5

^a^ Ref [9]; ^b^ Ref [16]; ^c^ Ref [35]; ^d^ Ref [11], ^e^ Ref [32], ^f^ Ref [21]; ^g^ present work; ^h^ 3- + 15-a-DON.

**Table 4:** Mycotoxins (μg/kg) detected in fish feed samples (*n* = 3).

	1	2	3	4	5	6	7	8	9
15-a-DON			55 ± 2			21 ± 3			<LOQ
FB1	<LOQ	<LOQ			<LOQ	136 ± 16			<LOQ
FB2	<LOQ	<LOQ	<LOQ	<LOQ	<LOQ	105 ± 1	<LOQ	<LOQ	<LOQ
ZEN					<LOQ	121 ± 14			
ENNB	1.1 ± 0.1	1.6 ± 0.1	21 ± 1	0.94 ± 0.02	6.1 ± 1.0	6.5 ± 0.6	0.6 ± 0.1	2.6 ± 0.2	3.1 ± 0.1
BEA	6.6 ± 0.1	0.56 ± 0.04	0.5 ± 0.1	6.5 ± 0.3	9.4 ± 1.0	30 ± 2	0.9 ± 0.1	0.54 ± 0.03	16 ± 0.5
ENNB1	0.7 ± 0.2	1.5 ± 0.7	7.9 ± 1.2	0.7 ± 0.3	3.4 ± 0.2	2.3 ± 0.9	1.9 ± 0.8	2.4 ± 0.2	1.8 ± 0.3
ENNA1		<LOQ	1.9 ± 0.5			0.5 ± 0.1		0.47 ± 0.01	

**Table 5:** Optimized MRM conditions for the analysis of the selected mycotoxins.

Compound	MRM 1	CE (eV)	MRM 2	CE (eV)	Fragmentor (V)	Polarity
NIV	357.1 > 45	42	357.1 > 281.1	22	150	Negative
DON	297.1 > 249.1	10	297.1 > 203.1	10	100	Positive
15-a-DON	356.1 > 137.1	15	356.1 > 261.2	15	90	Positive
3-a-DON	339.1 > 231.1	10	339.1 > 213.1	20	110	Positive
AFB2	315.2 > 287	24	315.2 > 259	28	190	Positive
AFB1	313 > 285.2	20	313.2 > 259	38	190	Positive
FB1	722.5 > 334.4	44	722.5 > 352.3	36	210	Positive
FB2	706.3 > 336.3	40	706.3 > 318.5	40	220	Positive
OTA	404 > 239	20	404.1 > 221	36	115	Positive
ZEN	317 > 131	28	317 > 175	20	195	Negative
ENNB	657 > 196	32	657 > 214	32	160	Positive
BEA	801.5 > 244	36	801.5 > 262	32	180	Positive
ENNB1	672 > 196	32	671.4 > 214	60	170	Positive
ENNA1	685 > 210	32	685 > 228	32	150	Positive
ENNA	699 > 210	32	699 > 228	32	170	Positive

CE = collision energy.

## Data Availability

Data is contained within the article.

## References

[1] FAO (2017). The Future of Food and Agriculture: Trends and Challenges.

[2] FAO (2020). The State of World Fisheries and Aquaculture 2020. Sustainability in Action.

[3] Nácher-Mestre J., Beltrán E., Strachan F., Dick J.R., Pérez-Sánchez J., Berntssen M.H.G., Tocher D.R. (2020). No transfer of the non-regulated mycotoxins, beauvericin and enniatins, from feeds to farmed fish reared on plant-based diets. Food Chem..

[4] Pereira C.S., Cunha S.C., Fernandes J.O. (2019). Prevalent mycotoxins in animal feed: Occurrence and analytical methods. Toxins.

[5] Oliveira M., Vasconcelos V. (2020). Occurrence of mycotoxins in fish feed and its effects: A review. Toxins.

[6] Gonçalves R.A., Schatzmayr D., Albalat A., Mackenzie S. (2020). Mycotoxins in aquaculture: Feed and food. Rev. Aquac..

[7] Directive 2002/32/EC of the European Parliament and of the Council of 7 May 2002 on Undesirable Substances in Animal Feed—Council Statement. http://data.europa.eu/eli/dir/2002/32/oj.

[8] Commission Recommendation of 17 August 2006 on the Presence of Deoxynivalenol, Zearalenone, Ochratoxin A, T-2 and HT-2 and Fumonisins in Products Intended for Animal Feeding. http://data.europa.eu/eli/reco/2006/576/oj.

[9] Tebele S.M., Gbashi S., Adebo O., Changwa R., Naidu K., Njobeh P.B. (2020). Quantification of multi-mycotoxin in cereals (maize, maize porridge, sorghum and wheat) from Limpopo province of South Africa. Food Addit. Contam. Part A.

[10] Varga E., Glauner T., Berthiller F., Krska R., Schuhmacher R., Sulyok M. (2013). Development and validation of a (semi-)quantitative UHPLC-MS/MS method for the determination of 191 mycotoxins and other fungal metabolites in almonds, hazelnuts, peanuts and pistachios. Anal. Bioanal. Chem..

[11] Kim D.B., Song N.E., Nam T.G., Lee S., Seo D., Yoo M. (2019). Occurrence of emerging mycotoxins in cereals and cereal-based products from the Korean market using LC-MS/MS. Food Addit. Contam. Part A.

[12] Rausch A., Brockmeyer R., Schwerdtle T. (2020). Development and Validation of a QuEChERS-Based Liquid Chromatography Tandem Mass Spectrometry Multi-Method for the Determination of 38 Native and Modi fi ed Mycotoxins in Cereals. J. Agric. Food Chem..

[13] Anater A., Manyes L., Meca G., Ferrer E., Luciano F.B., Pimpão C.T., Font G. (2016). Mycotoxins and their consequences in aquaculture: A review. Aquaculture.

[14] Scarpino V., Reyneri A., Blandino M. (2019). Development and comparison of two multiresidue methods for the determination of 17 Aspergillus and *Fusarium mycotoxins* in cereals using HPLC-ESI-TQ-MS/MS. Front. Microbiol..

[15] Juan C., Oueslati S., Mañes J., Berrada H. (2019). Multimycotoxin determination in Tunisian farm animal feed. J. Food Sci..

[16] Dagnac T., Latorre A., Fernández Lorenzo B., Llompart M. (2016). Validation and application of a liquid chromatography-tandem mass spectrometry based method for the assessment of the co-occurrence of mycotoxins in maize silages from dairy farms in NW Spain. Food Addit. Contam. Part A.

[17] Albero B., Sánchez-Brunete C., Miguel E., Tadeo J.L. (2017). Application of matrix solid-phase dispersion followed by GC–MS/MS to the analysis of emerging contaminants in vegetables. Food Chem..

[18] Albero B., Tadeo J.L., Pérez R.A. (2020). Determination of emerging contaminants in cereals by gas chromatography-tandem mass spectrometry. Front. Chem..

[19] Pereira V.L., Fernandes J.O., Cunha S.C. (2014). Mycotoxins in cereals and related foodstuffs: A review on occurrence and recent methods of analysis. Trends Food Sci. Technol..

[20] De Boevre M., Di Mavungu J.D., Maene P., Audenaert K. (2012). Development and validation of an LC-MS/MS method for the simultaneous determination of deoxynivalenol, zearalenone, T-2-toxin and some masked metabolites in different cereals and cereal-derived food. Food Addit. Contam. Part A.

[21] Tolosa J., Font G., Mañes J., Ferrer E. (2014). Natural occurrence of emerging Fusarium mycotoxins in feed and fish from aquaculture. J. Agric. Food Chem..

[22] EFSA Panel on Contaminants in the Food Chain (CONTAM) (2014). Panel Scientific Opinion on the risks to human and animal health related to the presence of beauvericin and enniatins in food and feed. Efsa J..

[23] Lhotská I., Gajdošová B., Solich P., Šatínský D. (2018). Molecularly imprinted vs. reversed-phase extraction for the determination of zearalenone: A method development and critical comparison of sample clean-up efficiency achieved in an on-line coupled SPE chromatography system. Anal. Bioanal. Chem..

[24] López-Blanco R., Nortes-Méndez R., Robles-Molina J., Molina-Díaz A. (2016). Evaluation of different cleanup sorbents for multiresidue pesticide analysis in fatty vegetable matrices by liquid chromatography tandem mass spectrometry. J. Chromatogr. A.

[25] Slámová T., Sadowska-Rociek A., Fraňková A., Surma M., Banout J. (2020). Application of QuEChERS-EMR-Lipid-DLLME method for the determination of polycyclic aromatic hydrocarbons in smoked food of animal origin. J. Food Compos. Anal..

[26] Lucas D., Zhao L. (2017). Multiclass Mycotoxin Analysis in Cheese Using Agilent Captiva EMR—Lipid Cleanup and LC/MS/MS.

[27] Lucas D. (2019). Mycotoxin Analysis in Infant Formula Using Captiva EMR—Lipid Cleanup and LC/MS/MS.

[28] Arce-López B., Lizarraga E., Flores-Flores M., Irigoyen Á., González-Peñas E. (2020). Development and validation of a methodology based on Captiva EMR-lipid clean-up and LC-MS/MS analysis for the simultaneous determination of mycotoxins in human plasma. Talanta.

[29] Nakhjavan B., Ahmed N.S., Khosravifard M. (2020). Development of an improved method of sample extraction and quantitation of multi-mycotoxin in feed by LC-MS/MS. Toxins.

[30] Stroka J., Petz M., Joerissen U., Anklam E. (1999). Investigation of various extractants for the analysis of aflatoxin B1 in different food and feed matrices. Food Addit. Contam..

[31] Li H., Wang C., Zhu Q., Du H., Guan S., Wang F., Zhang W., Fan W., Chen Z., Yang G. (2016). Reduction of matrix effects through a simplified QuEChERS method and using small injection volumes in a LC-MS/MS system for the determination of 28 pesticides in fruits and vegetables. Anal. Methods.

[32] Mwihia E.W., Lyche J.L., Mbuthia P.G., Ivanova L., Uhlig S., Gathumbi J.K., Maina J.G., Eshitera E.E., Eriksen G.S. (2020). Co-Occurrence and levels of mycotoxins in fish feeds in Kenya. Toxins.

[33] Konak I., Yatmaz H.A., Nilüfer, Erkaymaz T., Certel M. (2021). Multiresidue method for the simultaneous analysis of antibiotics and mycotoxins in feeds by ultra-high performance liquid chromatography coupled to tandem mass spectrometry. Acta Aliment..

[34] Bernal E. (2014). Limit of detection and limit of quantification determination in gas chromatography. Adv. Gas Chromatogr..

[35] Jedziniak P., Pietruszka K., Burek O., Wiśniewska-Dmytrow H. (2016). Mycotoxin determination in animal feed by UPLC-MS/MS Development of a UPLC-MS/MS method for determination of mycotoxins in animal Feed. Euroreference.

[36] Bernal-Algaba E., Pulgarín-Alfaro M., Fernández-Cruz M.L. (2021). Cytotoxicity of mycotoxins frequently present in aquafeeds to the fish cell line RTGill-W1. Toxins.

[37] Fegan D.F., Spring P. (2007). Recognizing the reality of the aquaculture mycotoxin problem: Searching for a common and effective solution. Nutritional Biotechnology in the Feed and Food Industries, Proceedings of Alltech’s 23rd Annual Symposium. The New Energy Crisis: Food, Feed or Fuel, Lexington, KY, USA, 20–23 May 2007.

[38] Woźny M., Obremski K., Jakimiuk E., Gusiatin M., Brzuzan P. (2013). Zearalenone contamination in rainbow trout farms in north-eastern Poland. Aquaculture.

[39] Nácher-Mestre J., Serrano R., Beltrán E., Pérez-Sánchez J., Silva J., Karalazos V., Hernández F., Berntssen M.H.G. (2015). Occurrence and potential transfer of mycotoxins in gilthead sea bream and Atlantic salmon by use of novel alternative feed ingredients. Chemosphere.

